# Retraction Note: Origin and cross-species transmission of bat coronaviruses in China

**DOI:** 10.1038/s41467-024-55454-w

**Published:** 2024-12-19

**Authors:** Alice Latinne, Ben Hu, Kevin J. Olival, Guangjian Zhu, Libiao Zhang, Hongying Li, Aleksei A. Chmura, Hume E. Field, Carlos Zambrana-Torrelio, Jonathan H. Epstein, Bei Li, Wei Zhang, Lin-Fa Wang, Zheng-Li Shi, Peter Daszak

**Affiliations:** 1 EcoHealth Alliance, New York, USA; 2 Key Laboratory of Special Pathogens And Biosafety, Wuhan Institute of Virology, Center for Biosafety Mega-Science, Chinese Academy of Sciences, Wuhan, China; 3 Guangdong Institute of Applied Biological Resources, Guangdong Academy of Sciences, Guangzhou, China; 4 School of Veterinary Science, The University of Queensland, Brisbane, QLD Australia; 5 Programme in Emerging Infectious Diseases, Duke-NUS Medical School, Singapore, Singapore; 6 Present Address: Wildlife Conservation Society, Viet Nam Country Program, Ha Noi, Viet Nam; Wildlife Conservation Society, Health Program, Bronx, NY USA

**Keywords:** Phylogenetics, Evolutionary biology, SARS-CoV-2, Viral evolution

Retraction to: *Nature Communications* 10.1038/s41467-020-17687-3, published online 25 August 2020

The Authors have retracted this article.

Following publication, errors in the sequence data included in the analyses in the article were identified. Out of 1246 sequences analysed, 41 coronavirus (CoV) sequences (12 alpha-CoVs and 29 beta-CoVs) obtained from bats sampled in the border region of northern Lao PDR (Luang Namtha province) were erroneously included in the analyses. These sequences will remain available on GenBank under the following accession codes: MN312304-MN312315, MN312608-MN312631, and MN312666-MN312670. Additionally, 27 duplicated sequences were erroneously included in the analyses.

A revised article reports the analyses with the Laotian and duplicate sequences removed. It has undergone peer review independently from the original article’s review process and has been published^1^.

All Authors agree with this retraction.
